# Epileptic Seizures Prediction Using Machine Learning Methods

**DOI:** 10.1155/2017/9074759

**Published:** 2017-12-19

**Authors:** Syed Muhammad Usman, Muhammad Usman, Simon Fong

**Affiliations:** ^1^Shaheed Zulfikar Ali Bhutto Institute of Science and Technology, Islamabad, Pakistan; ^2^University of Macau, Macau

## Abstract

Epileptic seizures occur due to disorder in brain functionality which can affect patient's health. Prediction of epileptic seizures before the beginning of the onset is quite useful for preventing the seizure by medication. Machine learning techniques and computational methods are used for predicting epileptic seizures from Electroencephalograms (EEG) signals. However, preprocessing of EEG signals for noise removal and features extraction are two major issues that have an adverse effect on both anticipation time and true positive prediction rate. Therefore, we propose a model that provides reliable methods of both preprocessing and feature extraction. Our model predicts epileptic seizures' sufficient time before the onset of seizure starts and provides a better true positive rate. We have applied empirical mode decomposition (EMD) for preprocessing and have extracted time and frequency domain features for training a prediction model. The proposed model detects the start of the preictal state, which is the state that starts few minutes before the onset of the seizure, with a higher true positive rate compared to traditional methods, 92.23%, and maximum anticipation time of 33 minutes and average prediction time of 23.6 minutes on scalp EEG CHB-MIT dataset of 22 subjects.

## 1. Introduction

The disease in which patients suffer seizures caused by a brain functionality disorder is called epilepsy [1]. While more than fifty million people around the world are diagnosed with epilepsy [2], in the United States, about three million patients have been affected by epilepsy. Epilepsy is the third most common brain disorder [3]. Meanwhile, there are several possible causes of epilepsy, one of which is a molecular mutation, which results in irregular neuronal behavior or migration of neurons. Although the main cause of epilepsy remains unknown, early diagnosis can be useful for treating epilepsy. Epilepsy patients can be treated with drugs or surgical procedures [4]. However, these methods are not fully effective. Unfortunately, seizures that cannot be completely treated medically limit the active life of the patient. In these cases, patients cannot independently work and do some activity. This leads to social isolation of individuals and economic difficulties.

**Figure 1:**
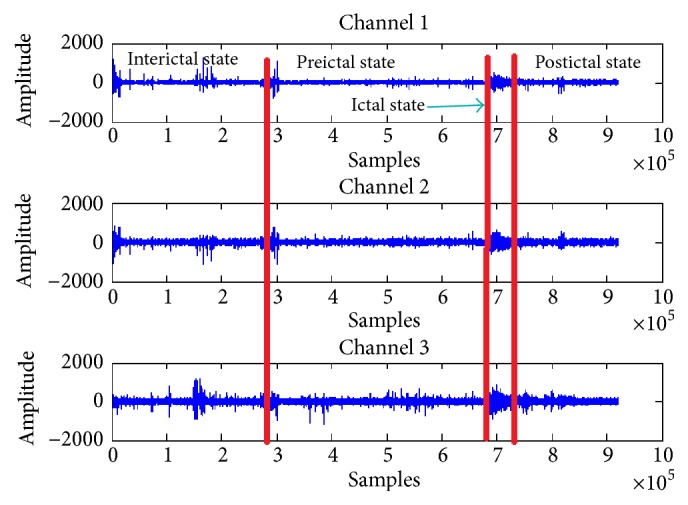
States of epileptic seizure.

Early prediction of epileptic seizures ensures enough time before it actually occurs; it is very useful because the attack can be avoided by the drug. Epileptic seizures have four different states: the preictal state, which is a state that appears before the seizure begins, the ictal state that begins with the onset of the seizure and ends with an attack, the postictal state that starts after ictal state, and interictal state that starts after the postictal state of 1st seizure and ends before the start of preictal state of consecutive seizure.  Figure 1 shows different input states of three different channels. In addition, seizures can be predicted by detecting the beginning of the preictal state.

Detecting the appearance of preictal state [5] predicts the seizure. Therefore, the purpose of our investigation is to detect the appearance of preictal state for epileptic seizures. Machine learning models are used to predict epileptic seizures. These machine learning models include EEG signal acquisition, signal preprocessing, features extraction from the signals, and finally classification [6] between different seizure states. The objective of the prediction model with machine learning was to detect preictal state's sufficient time before seizure onset starts [7]. However, enough time for the predictive preictal state of the gland and maximum sensitivity are important, and they remain as a performance issue in the prediction of epileptic seizures.

Preprocessing and feature extraction from EEG signal have great affect in maximizing prediction time and true positive rate (TPR). Preprocessing is performed for removing noise from the signals and to increase the signal-to-noise ratio (SNR). Many researchers [8] have discussed preprocessing steps that include converting multiple channels of EEG signals into a single surrogate channel, and then filters have been applied to increase the signal-to-noise ratio (SNR). EEG signals that have been acquired by using multiple electrodes can be converted into surrogate channel by using averaging filter, common spatial filtering (CSP), large Laplacian filter, and optimized spatial pattern (OSP) filtering [8]. Many researchers have also discussed extraction of linear and nonlinear features for prediction of epileptic seizures. Features that distinguish between preictal and interictal states include autoregressive coefficients, relative power from EEG signals of different frequency bands, complexity, Hjorth parameters [9], zero crossing, Lyapunov exponent [10], and spectral and statistical moments [11].

Finally, as we have observed in the literature [12–16] that there is no machine learning model that provides an absolutely reliable method for both preprocessing and features extraction, we propose an effective and reliable machine learning model for prediction of epileptic seizures. Our model focuses on preprocessing and features extraction from EEG signals. We have converted multiple channels EEG signal into the surrogate signal, and then empirical mode decomposition (EMD) [17] has been applied for increasing signal-to-noise ratio (SNR). We have extracted multiple features including entropy, approximate entropy, Hjorth parameters, spectral moments, and statistical moments. It has been observed that both statistical and spectral features give increased sensitivity between interictal and preictal states. Support Vector Machine (SVM) [18] has been used as a classifier for classification between preictal state and interictal state.

The rest of the paper is organized as follows: Section 2 discusses related work.  Section 3 explains the proposed methodology.  Section 4 reports and compares the experimental results.  Section 5 concludes the paper and mentions future work.

## 2. Related Work

In the previous section, we have discussed four states of seizures. Among these four states, ictal state and preictal state are very useful for predicting epileptic seizures. The ictal state can be used to classify seizure and nonseizure EEG signals. The preictal state is another useful state of epileptic seizures. It begins several minutes before the onset of a seizure, and it ends with the start of ictal state. Many researchers [12–16] tried to detect the beginning of the preictal state by using EEG signals. However, only a few have reliably detected the preictal state of epilepsy. Preprocessing of the EEG signal to increase signal-to-noise ratios and features extraction play an important role in reliable prediction of epileptic seizures.

The combination of multiple unique features into a feature vector can be used to predict the preictal state of epileptic seizures. Rasekhi et al. in [12] have proposed an algorithm for seizure prediction with the help of univariate linear features. In [12], the authors used only six EEG channels in their proposed model and extracted 22 univariate linear properties. Thus, a 132-dimensional feature space is created. It was assumed that preictal time starts 10 to 40 minutes before the ictal state with a difference of 10 minutes. Prediction of epileptic seizures is considered by classifying a binary class that classifies test data into either preictal state or ictal state. On average, the prediction sensitivity after applying this algorithm is 73.90%.

In [12], the authors used Support Vector Machine as a classifier to classify the preictal and ictal states of EEG signals. The authors have used extracted univariate linear features using the window size of seconds in their algorithm. In the second step, preprocessing is done, and finally a decision was made on EEG signal, following certain regularization. Three EEG channels have been extracted by placing electrodes on the patient's scalp focusing on seizure, while the three electrodes are located outside the confiscated surface. The data of the acquired EEG signals are converted into segments of a nonoverlapping window having the size of 5 seconds. After converting this data to the 5-second segments, the Butterworth filter [19] was used to reduce the noise effect.

The authors in [12] extracted the first four statistical moments as features. All these four features measure similarity, variance, and symmetry of consecutive samples of EEG signals. In order to deal with outliers, the authors have standardized all the features, so there will be no outliers. Although the noise has been reduced from signals, still there was some noise in the EEG signals, as the brain is a nonstationary source for recording the EEG signals. Smoothing is performed on the EEG signals to eliminate the noise.

It has been observed in literature that univariate linear features have better sensitivity performance for epilepsy data of EEG signals. Teixeira et al. in [13] have proposed a model for prediction of epileptic seizures by choosing only six channels of EEG signals and have extracted 22 linear univariate features for each channel. The overall feature space expands to 132 dimensions. In [13], the authors have used only six electrodes for EEG data acquisition. The main reason behind this minimum electrode selection is to set free the patient from wearing a large number of electrodes, as patients are often unwilling to wear so many electrodes on their scalp due to discomfort. Therefore, in order to give comfort to the mind, only six channels have been acquired and used for prediction purpose. The authors have selected these electrodes by using three different approaches.

One method is by randomly selecting six EEG electrodes, while the second method is to choose six channels from electrodes which have been placed on the scalp area from where seizures originate. The authors in [13] have used notch filter [20] for smoothing as preprocessing step for noise removal. They have also tested their model for prediction by varying multiple combinations of electrodes and also with four different preictal state durations. They have used three classifiers for classification and have approximately predicted every seizure.

After selecting suitable features, training data is fed into Support Vector Machine for training the classifier, and then test data is passed for determining classification accuracy and sensitivity. The authors have observed sensitivity of 75.8% of detecting the seizure, meaning that, out of 87, they have successfully detected 66 seizures. The authors have also proposed that performance can be improved by further reducing features set.

In [14], Bandarabadi et al. have proposed an algorithm to predict epilepsy seizures that can extend the life of epilepsy-affected patients. They have extracted spectral power features, and after suitable selection of features, features are passed into Support Vector Machines for classification. They have observed sensitivity of 75.8%; it means that their classifier has predicted 66 seizures out of total 87. They have concluded that, by applying these methods, after reducing proposed features subset can improve seizure prediction performance.

In [15], the authors have used wavelet method for prediction of seizures. They have extracted features including wavelet energy and wavelet entropy. Two or three channels have been selected for testing purposes on a dataset of six patients. Sensitivity has been reported as 88% with average anticipation time of 22 minutes.

 Zandi et al. [16] have also proposed a model for predicting seizures using scalp EEG signals on the basis of zero crossings. The authors in [16] have computed the histogram of all intervals in a moving average window and have selected values from particular bins for observations. Once the whole process is completed, last 5 seconds of observations are compared with different reference sets of points, containing preictal and interictal states. They have measured a similarity index on the basis of variational Bayesian Gaussian mixture [21] model of EEG data.

A combined similarity index is calculated after measuring the similarity index on the basis of a particular threshold. An alarm is generated upon indication of the start of the preictal state of the seizure which predicts the seizure. The authors have applied their model for epileptic seizure prediction on a dataset of 20 subjects including 86 seizures. They have observed sensitivity of the model of 88.3%. Average anticipation time in their case, that is, the time of detecting preictal state before onset of the seizure, has been observed to be 22.5 minutes.  Table 1 shows a comparison of various machine learning models on the basis of the number of subjects, number of seizures, type of EEG data, sensitivity, and false positive rates.

It has been observed in researches of Table 1 that there is no reliable method for selection of channels from EEG signals, and no reliable preprocessing method has been devised. Therefore, keeping in mind the fact that preprocessing of the EEG signals can improve prediction sensitivity and average anticipation time, we propose an effective machine learning method for epilepsy prediction.

## 3. Proposed Method

The preictal state is very useful for seizure prediction, as it starts few minutes before the seizure. This made it possible for us to be able to predict epileptic seizure, if we successfully detect the start of preictal state. The aim of this research is to predict epileptic seizure by detecting the start of preictal state's sufficient time before the ictal state or onset of seizure starts. Early prediction [22] helps patients, as medication can be done by the doctors to prevent the seizure. Due to this medication, the patient can now perform his or her routine activities without any interference from seizures. After critical considerations of these states, we have proposed a model to detect the start of the preictal state. However, EEG data acquisition by placing electrodes on the scalp of the patient is out of the scope of our research. Consequently, we have used a publically free online available dataset of CHB-MIT [23]. The dataset has been acquired by placing 23 electrodes on the scalp of 22 subjects. We have performed preprocessing of the data in two stages; in the first stage, 23 channels EEG signals are converted into a surrogate channel, which is a single signal to improve the SNR. In the second stage of preprocessing, empirical mode decomposition (EMD) has been applied to the surrogate channel for further increasing SNR.

It is pertinent to know that the surrogate channel of EEG signal can be obtained by applying either averaging filter, large Laplacian filter, or common spatial pattern (CSP) filtering [8]. Since we do not have any information about the reference electrode, among the 23 electrodes, large Laplacian filter is not possible, as it requires information about reference electrode for assigning maximum weight to it. We have applied averaging filter and CSP to obtain surrogate channel and have concluded after comparing results that CSP gives increased SNR; CSP also increases within a class variance of signals.

We have performed the empirical mode decomposition (EMD) after converting into the surrogate channel to increase the signal-to-noise ratio. Empirical mode decomposition decomposes the surrogate channel EEG signal into its oscillatory functions, known as Intrinsic Mode Functions (IMFs). Noise affects high-frequency components; therefore, we have taken only last four IMFs having low frequencies and contained more information about seizures. These four IMFs are combined, and the features have been extracted for classification.

Features have been extracted in both time and frequency domains. Statistical features have been extracted in a time domain, whereas spectral features have been extracted in a frequency domain. We have compared three classifiers in terms of sensitivity, and Support Vector Machine has been selected as it gives greater sensitivity; therefore, classification is done by using Support Vector Machines in order to distinguish testing data between preictal and interictal states.  Figure 4 shows a flowchart that illustrates our proposed method.

**Figure 2:**
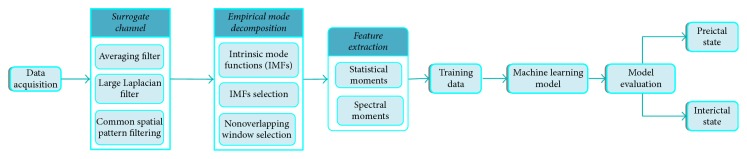
Flowchart for epilepsy prediction.

**Figure 3:**
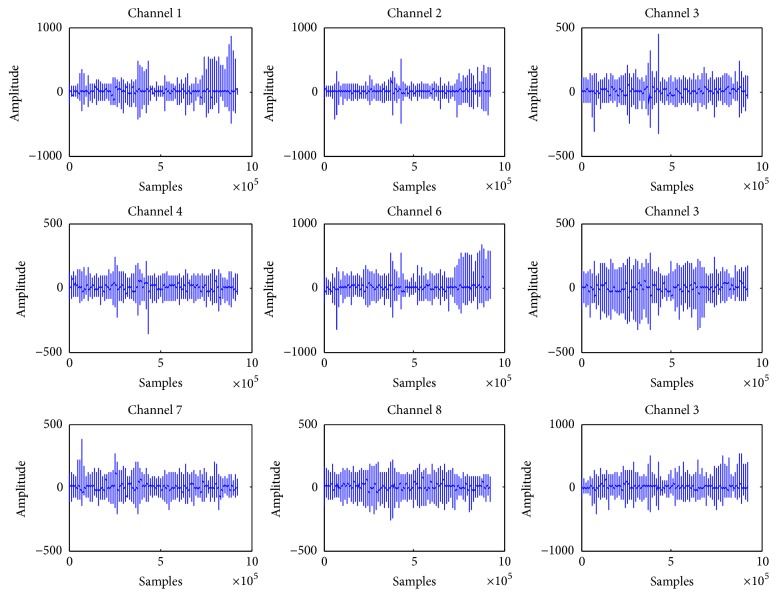
Single channel EEG signal.

Figure 2 shows our proposed flowchart. Meanwhile, data acquisition has been performed in the first step by placing electrodes on the scalp of subjects, and data is stored in edf format [24]. edf data is converted into MATLAB [21] files having the extension ^*∗*^.mat with the help of MATLAB function called “edfread.” Figure 3 shows plots of different channels of EEG signal for a single session. Most of the sessions are of one-hour recordings, as data has been acquired using 23 electrodes and the sampling rate of 256 Hz. Noise has been added in 23 channels of EEG signals during the recording of these signals; therefore, in order to increase the signal-to-noise ratio (SNR) of the signal, we have to convert it into a surrogate channel by applying common spatial pattern (CSP) filtering. In the next step, empirical mode decomposition (EMD) is applied on the surrogate channel EEG signal for further increasing SNR. Statistical features in time domain and spectral features in frequency domain have been extracted.

### 3.1. Surrogate Channel

CHB-MIT dataset has been acquired by placing 23 electrodes on the scalp of subjects; therefore 23 channels have been obtained. These channels contain noise that affects prediction directly; therefore, we have to convert these channels into a surrogate channel with increased SNR. The multiple channels signal can be converted into a single surrogate channel by applying common averaging filter, large Laplacian spatial filter, and common spatial pattern (CSP) filter.

#### 3.1.1. Averaging Filter

**Figure 4:**
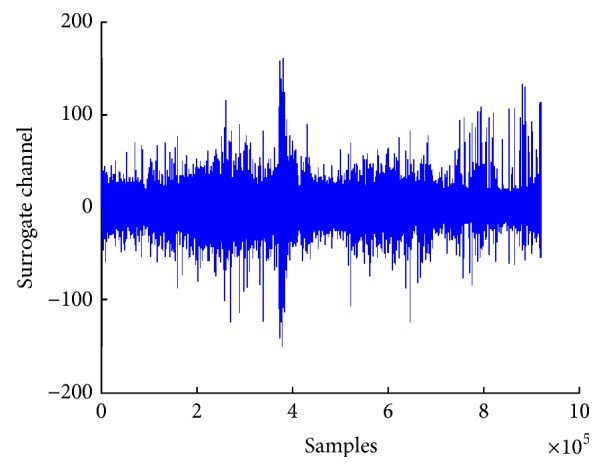
Surrogate channel after applying averaging.

We have applied a simple averaging filter on the multiple channels EEG single to convert it into the surrogate channel. This filter computes the average of all channels to form a single-channel EEG signal. Although the surrogate channel obtained after applying this filter has more signal-to-noise ratio than multiple channels signal, there is still some noise in it.  Figure 4 shows plot of surrogate channel EEG signal obtained after applying averaging filter.

#### 3.1.2. Large Laplacian Filter

Following our observation, averaging filter has not given better results. Large Laplacian filter also gives increased signal-to-noise ratio and can be used for surrogate channel [25]. In our case, CHB-MIT dataset has no information about reference electrode, and, without this information, we cannot apply large Laplacian filter.

#### 3.1.3. Common Spatial Pattern Filter

Common spatial pattern (CSP) filter [26] can be used to convert multiple channels signal into the surrogate channel, as it gives increased signal-to-noise ratio. It also performs better in case of EEG signals as it increases SNR and the variance interval between two class variables. If *X*_1_ and *X*_2_ are the signals from two different epilepsy states, that is, preictal state and interictal state, then you get *R*_1_ and *R*_2_ when you divide the squares of signals by the trace in (1) and (2), respectively.

(1) R1=X1X1ttraceX1X1t

(2) R2=X2X2ttraceX2X2t

(3) R=R1+R2

(4) Evec,Eval=eigR.

*R*_1_ and *R*_2_ are added in (3) and eigenvalue decomposition is performed in (4) which results in eigenvalues Eval and eigenvector Evec. Suppose that *D* is a matrix that contains all diagonal elements of eigenvector.

(5) w=D−1Evect

(6) S1=wR1wt

(7) S2=wR2wt

(8) B,D=eigS1,S2.

Weight *w* is computed in (5) and *S*_1_ and *S*_2_ are computed in (6) and (7). Eigenvalue decomposition is performed in (8) to get eigenvector *B* and eigenvalues *D*. These values are sorted in descending order in order to get a filter in the following equation:

(9) $$\begin{array}{c} Filter=\beta^{t}w. \end{array}$$

Equation (9) shows coefficients of common spatial pattern (CSP) filter. We have applied CSP on all sessions to get surrogate channel EEG signal in order to get increased SNR and high variance between classes of preictal and interictal states.

**Figure 5:**
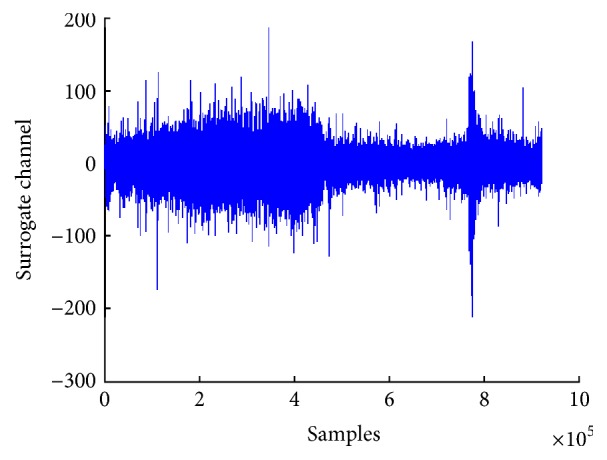
Surrogate channel after applying CSP.

Figure 5 shows a plot of EEG signal after applying CSP for converting it into a single surrogate channel. The plot shows that this surrogate channel indicates the start of preictal state.

### 3.2. Empirical Mode Decomposition (EMD)

In empirical mode decomposition (EMD) [27], a time domain signal is broken into a number of oscillatory functions known as Intrinsic Mode Functions (IMFs). This process of decomposition of signal into multiple IMFs while remaining in time domain is comparable with wavelet decomposition and Fourier transform. EMD is a very useful process for analyzing signals that are nonstationary and not linear. When empirical mode decomposition is applied on surrogate channel EEG signal, it filters out noise from the original signal. High-frequency components contain noise, whereas low frequencies contain original signal and information about seizures.

There are different frequency components that are obtained as a result of applying empirical mode decomposition and Intrinsic Mode Functions (IMFs). Multiple IMFs are obtained depending upon the nature of EEG signal. Meanwhile, there is an important fact about this decomposition, where the length of each Intrinsic Mode Function is equal to the length of the surrogate channel signal. We have applied EMD on surrogate channels of each session for all subjects in MATLAB. We have analyzed different IMFs and have observed that the last four IMFs obtained after applying EMD contain maximum information about states of seizures. Therefore, we have combined the last four IMFs, which are used for feature extraction. Every Intrinsic Mode Function must follow the following conditions: (1) The total peak values and the count of zero crossing should ideally be the same or have a maximum difference of 1. (2) At any given point of signal, the average envelope value is defined by local maxima, and envelope defined by local minima is zero. Assume that *x*(*t*) is the given signal; all maxima and minima can be obtained by Algorithm 1.

### 3.3. Selecting a Nonoverlapping Window

CHB-MIT dataset has been sampled at 256 Hz; therefore we have selected a nonoverlapping window of 1 second consisting of 256 samples. Selection of window has been done for downsampling the data in order to increase processing speed.

### 3.4. Feature Extraction

Several features have been extracted in this work, but spectral and statistical moments perform better in terms of both anticipation time and sensitivity. Therefore, we have extracted the first four statistical moments and the three spectral moments [28].

#### 3.4.1. Statistical Moments

Four statistical moments obtained from IMFs are quite useful in classification of different states of epileptic seizures. These statistical moments give information of the distribution of samples including variation between samples, symmetry, and peaks. Analysis of statistical moments of IMFs of seizure and nonseizure sessions obtained after applying EMD shows that this information can be easily classified. If *x*_*i*_ represents combined IMFs of the signal and *N* denotes length of the IMF; then statistical moments can be computed by the following equations:

(10) μt=1N∑i=1Nxi−μt2σt=1N∑i=1Nxi−μt2βt=1N∑i=1Nxi−μtσt3,

where *N* is the total number of samples of length of IMF; *μ*_*t*_ is the mean, *σ*_*t*_ is the standard deviation, and *β*_*t*_ is skewness of the corresponding IMF.

**Figure 6:**
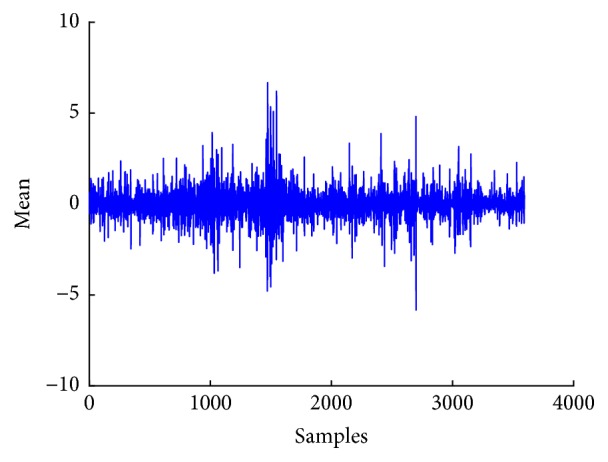
Mean of surrogate channel EEG signal.

**Figure 7:**
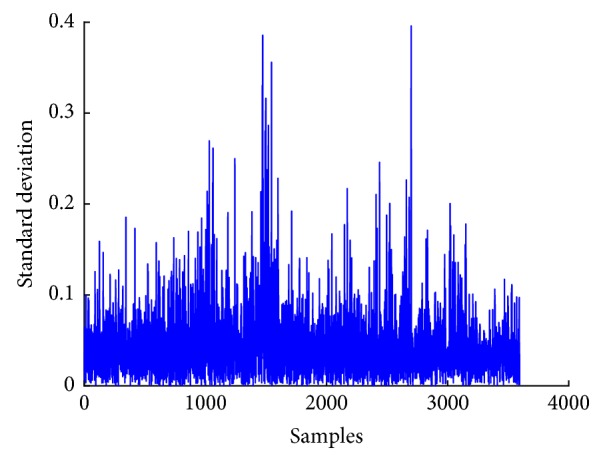
Standard deviation plot of EEG signal.

**Figure 8:**
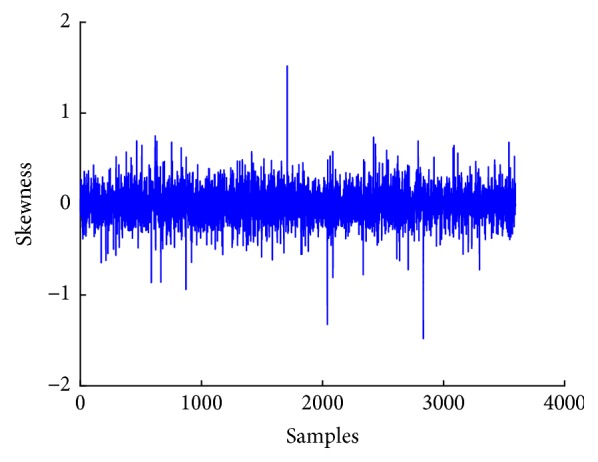
Skewness plot of EEG signal.

**Figure 9:**
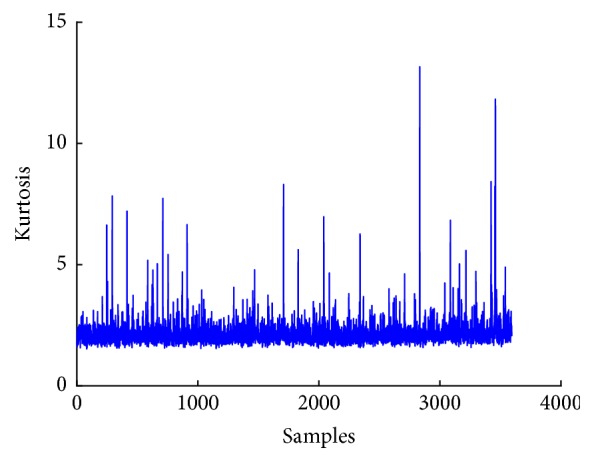
Kurtosis plot of EEG signal.

Figure 6 shows a plot of mean of EEG signal, whereas Figures 7, 8, and 9 show plots of standard deviation, skewness, and kurtosis for EEG signal, respectively.

#### 3.4.2. Spectral Features

Empirical mode decomposition can perform spectral analysis [29] for EEG signals. Amplitude of EEG signals increases in frequency domain during seizures, which is useful for prediction. Therefore, we have also extracted the features in frequency domain.

A conceptual understanding of empirical mode decomposition is that it decomposes a single signal into a number of Intrinsic Mode Functions (IMFs), which are obtained by passing from filters having narrow pass bands. Spectral moments have been extracted from Intrinsic Mode Functions (IMFs). These moments provide useful information of different states of seizures. Most of the time, when applying EMD, this spectral analysis is performed by using the calculation of instantaneous frequencies (IF). However, it is also reported that calculation of instantaneous frequencies has a physical meaning only for mono components of signal.

It is no longer news that when EMD is applied on EEG signals we never get monotonic component. As an alternative, we have computed power spectral density (PSD) [30] for extracting spectral moments. The discrimination power of the PSD features can be visually analyzed by their respective plots for three IMFs from the normal and pathological EEG signals. The PSD can be calculated as follows:

(11) $$\begin{array}{c} P\left(\;w\;\right)=\overset{N}{\underset{n=1}{\sum }}r_{y}\left[\;n\;\right]e^{-jwn}, \end{array}$$

where *r*_*y*_[*n*] represents the autocorrelation of *y*[*n*], defined as *r*_*y*_[*n*] = *E*(*y*[*m*]*y*[*m* + *τ*]).

However, autocorrelation is the correlation of the signal with itself and time delay. In the above equation, *y*[*m*] represents the signal and *y*[*m* + *τ*] represents its delayed version. Visual analysis of the PSD of IMFs shows that the statistics of the PSD can be used as relevant features for feature extraction.

**Figure 10:**
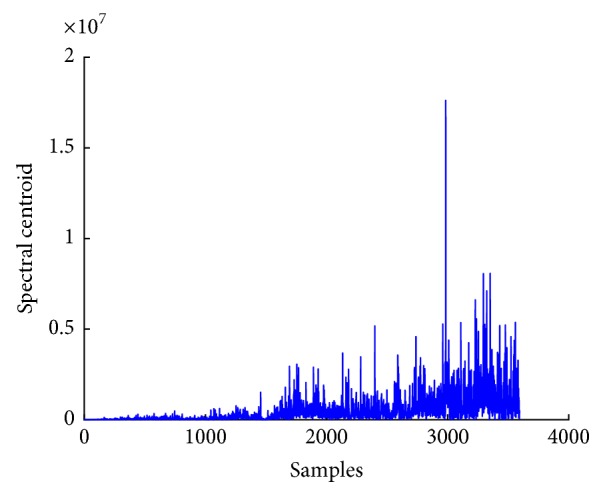
Spectral centroid plot of EEG signal.

*(1) Spectral Centroid*. As the spectral variation of the IMF differs for both normal and seizure subjects in EEG signals, the variational coefficient can be used as a feature for classification of EEG signals. Equation (13) gives the formula for computation of variational coefficient; *P*(*w*) is the amplitude of *w*th frequency bin in the spectrum. In (12), *C*_*s*_ computes spectral centroid of the signal.  Figure 10 shows plot of spectral centroid for EEG signal.

(12) $$\begin{array}{c} C_{s}=\frac{\sum_{w}^{}wP\left(w\right)}{\sum_{w}^{}P\left(w\right)}. \end{array}$$

**Figure 11:**
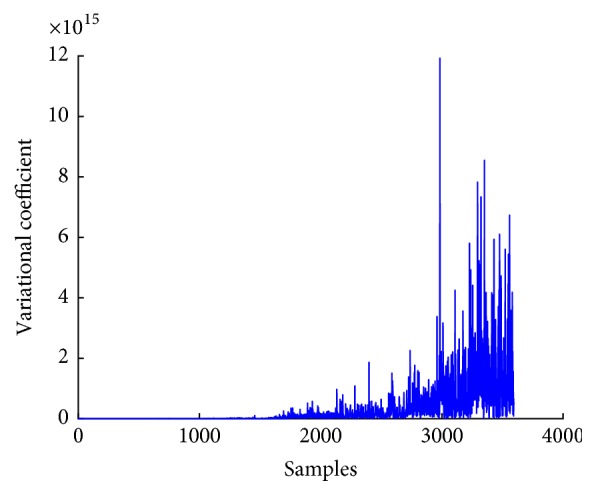
Variational coefficient plot of EEG signal.

**Figure 12:**
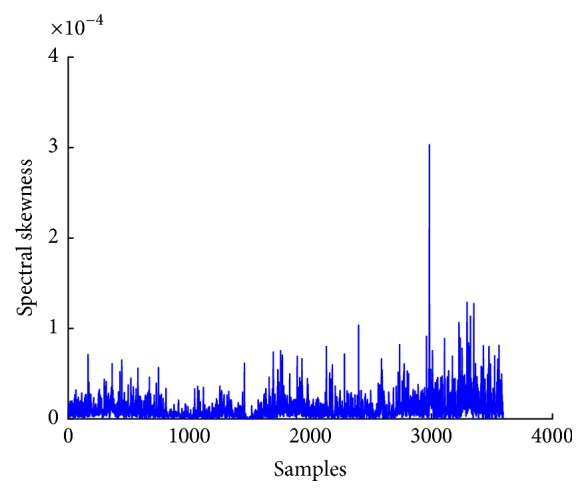
Spectral skewness plot of EEG signal.

*(2) Variational Coefficient*. As the spectral variation of the IMF differs for both normal and seizure subjects in EEG signals, variational coefficient can be used as feature for classification of EEG signals. Equation (13) gives the formula for computation of variational coefficient; *C*_*s*_ is the spectral centroid. Both *C*_*s*_ and power spectral density *P*(*w*) are used for computing variational coefficient *σ*_*s*_^2^.  Figure 11 shows plot of variational coefficient of EEG signal.

(13) $$\begin{array}{c} \sigma_{s}^{2}=\frac{\sum_{w}^{}{\left(w-C_{s}\right)}^{2}P\left(w\right)}{\sum_{w}^{}P\left(w\right)}. \end{array}$$

(*3*) *Spectral Skew*. Spectral skew is the third spectral moment, which measures the symmetry of the distribution of data. The plot of spectral skew of EEG signal shows that there is a significant change that occurs when in different states of seizure. Therefore, spectral skew can be a useful feature for prediction of epileptic seizure of EEG signal. Spectral skewness *β*_*s*_ can be computed by using spectral centroid *C*_*s*_, variational coefficient *σ*_*s*_, and power spectral density *P*(*w*) in (14).  Figure 12 shows plot of spectral skewness of EEG signal.

(14) $$\begin{array}{c} \beta_{s}=\frac{\sum_{w}^{}{\left(\left(w-C_{s}\right)/\sigma_{s}\right)}^{3}P\left(w\right)}{\sum_{w}^{}P\left(w\right)}. \end{array}$$

### 3.5. Combined Feature Set

First, four statistical moments and three spectral moments have been extracted from IMFs and combined into a single feature vector, which is used for classification.

### 3.6. Classification of Preictal and Interictal States

As we have already discussed that for predicting epileptic seizures it is important that we detect the start of preictal state, which shows that seizure is going to occur after few minutes, we need to classify preictal and interictal states of seizure. We have performed classification on features that we have extracted from the window of 1 second. The preictal state is detected where three samples are classified as preictal state samples.

### 3.7. Classifier Selection

We have extracted some temporal features from ictal and nonictal state and performed a comparison for suitable selection of a classifier.

**Figure 13:**
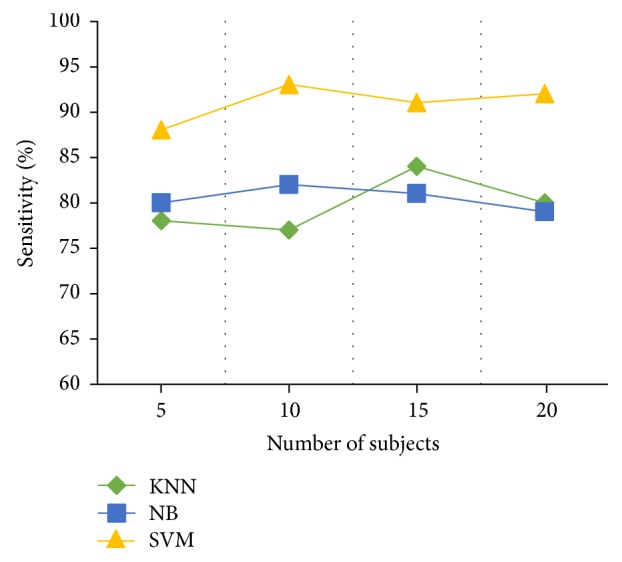
Comparison of classifiers.

Three classifiers have been applied: *k*-nearest neighbor classifier, naïve Bayes, and Support Vector Machines. After performing the comparison of three different classifiers, Support Vector Machine has been selected for prediction of epileptic seizures because it performs better in terms of sensitivity.  Figure 13 shows comparison of sensitivity of three different classifiers.

## 4. Results

For the selection of a suitable classifier, we have performed a classification of the ictal state with rest of states of seizure. Support Vector Machine is chosen as a classifier due to its superior performance in terms of sensitivity. After selection of classifier, we have applied our model for prediction of epileptic seizures on CHB-MIT dataset that contains EEG signals recordings of several hours for 24 patients of 3–19 years of age having 84 seizures, and the data is sampled at the sampling rate of 256 Hz. We have selected only those sessions from datasets which have seizure onset, at least after 20 minutes from start of the session.

We have computed average anticipation time for 84 sessions only from a complete CHB-MIT dataset. We have computed average prediction time of epileptic seizure as 23.48 minutes, whereas maximum prediction time has been observed as 33.46 minutes before the onset of the seizure. Average sensitivity has been observed as 92.23% and specificity as 93.38%.

**Figure 14:**
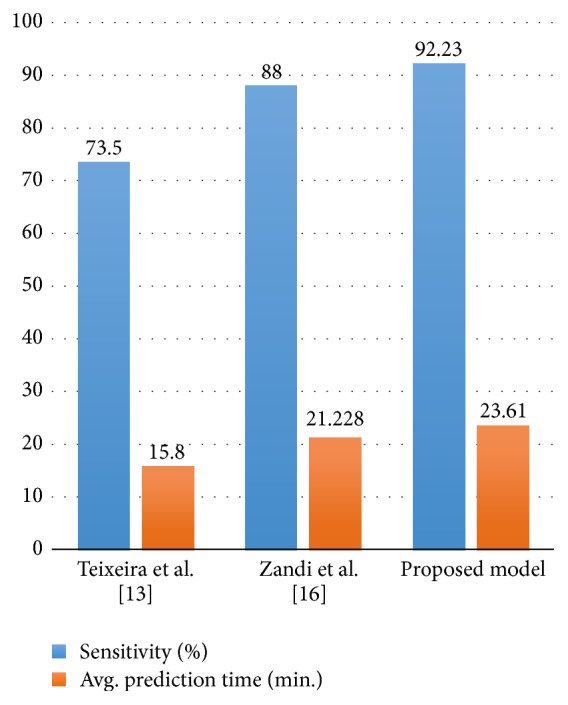
Comparison of prediction time and sensitivity.

Figure 14 and Table 2 show a comparison of results generated by our proposed model and those by other models. It is quite evident from the comparison that our model performs better in terms of both sensitivities and anticipation time as compared to Zandi et al. [16] and Teixeira et al. [13].

## 5. Conclusion and Future Work

In this research, we have used the CHB-MIT dataset that was recorded by placing electrodes on scalp of subjects to predict epileptic seizure. We have tested our proposed model on the dataset, and it has been shown in results that our model performs better in terms of both sensitivity and average prediction time as compared to other models for prediction of epileptic seizures. After applying the proposed model on the dataset, on average we have predicted epileptic seizures 23.6 minutes before the start of the onset of a seizure. Therefore, with the help of our proposed model, epilepsy-affected patients will get more time for proper medication required for preventing the seizure before it actually occurs. We have also observed maximum prediction time of 33.46 minutes on the dataset.

In the future, preprocessing of the EEG signal can be further improved to get an increased sensitivity of seizure prediction. Other preprocessing methods can be tried, including those hybrid preprocessing methods and those that come with adaptive window sizes [31]. Moreover, we can also develop an online system for prediction of epileptic seizures.

## Figures and Tables

**Algorithm 1:**
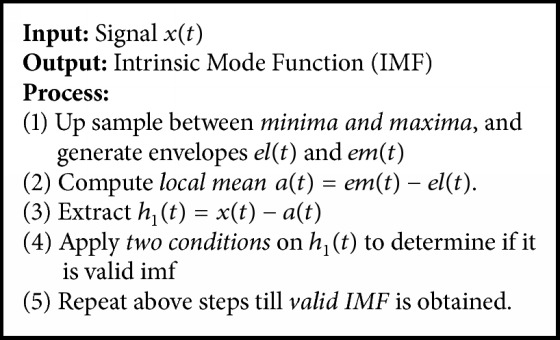


**Table 1:** Comparison of machine learning models for prediction of epileptic seizures.

Method	Number of subjects	Number of seizures	EEG channels used	Number of features	Average sensitivity (%)	FPR (*h*^−1^)
Rasekhi et al. [12]	10	86	6	22	71.97	0.17
Teixeira et al. [13]	224	87	6	22	73.08	0.33
Bandarabadi et al. [14]	24	87	6	12	73.98	0.06
Zandi et al. [16]	20	86	18/23	18/23	88.34	0.155
Gadhoumi et al. [15]	6	86	2/3	3	88	0.15

**Table 2:** Comparison of prediction time and sensitivity.

Model	Dataset	EEG signals type	Number of subjects	Number of seizures	Avg. prediction time (min.)	Sensitivity (%)
Teixeira et al. [13]	EPILEPSIAE	Scalp EEG	227	87	15.8	73.5
Zandi et al. [16]	VGH	Scalp EEG	17	86	21.48	91.11
	CHB-MIT	Scalp EEG	03		19.8	83.81
*Proposed model*	*CHB-MIT*	*Scalp EEG*	*24*	*84*	*23.61*	*92.23*

## References

[1] Acharya U. R., Sree S. V., Swapna G., Martis R. J., Suri J. S. (2013). Automated EEG analysis of epilepsy: a review. *Knowledge-Based Systems*.

[2] Fisher R. S., Van Emde Boas W., Blume W. (2005). Epileptic seizures and epilepsy: definitions proposed by the International League Against Epilepsy (ILAE) and the International Bureau for Epilepsy (IBE). *Epilepsia*.

[3] Hebert L. E., Scherr P. A., Bienias J. L., Bennett D. A., Evans D. A. (2003). Alzheimer disease in the US population: prevalence estimates using the 2000 census. *JAMA Neurology*.

[4] Guenot M. (2004). Surgical treatment of epilepsy: Outcome of various surgical procedures in adults and children. *Revue Neurologique*.

[5] Wang Y., Zhou W., Yuan Q. (2013). Comparison of ictal and interictal eeg signals using fractal features. *International Journal of Neural Systems*.

[6] Engel J. (2006). ILAE classification of epilepsy syndromes. *Epilepsy Research*.

[7] Moghim N., Corne D. W. (2014). Predicting epileptic seizures in advance. *PLoS ONE*.

[8] Ramoser H., Müller-Gerking J., Pfurtscheller G. (2000). Optimal spatial filtering of single trial EEG during imagined hand movement. *IEEE Transactions on Neural Systems and Rehabilitation Engineering*.

[9] Vidaurre C., Krämer N., Blankertz B., Schlögl A. (2009). Time domain parameters as a feature for EEG-based brain computer interfaces. *Neural Networks*.

[10] Osowski S., Swiderski B., Cichocki A., Rysz A. (2007). Epileptic seizure characterization by Lyapunov exponent of EEG signal. *COMPEL - The International Journal for Computation and Mathematics in Electrical and Electronic Engineering*.

[11] Chua K. C., Chandran V., Acharya U. R., Lim C. M. (2010). Application of higher order statistics/spectra in biomedical signals-A review. *Medical Engineering and Physics*.

[12] Rasekhi J., Mollaei M. R. K., Bandarabadi M., Teixeira C. A., Dourado A. (2013). Preprocessing effects of 22 linear univariate features on the performance of seizure prediction methods. *Journal of Neuroscience Methods*.

[13] Teixeira C. A., Direito B., Bandarabadi M. (2014). Epileptic seizure predictors based on computational intelligence techniques: A comparative study with 278 patients. *Computer Methods and Programs in Biomedicine*.

[14] Bandarabadi M., Teixeira C. A., Rasekhi J., Dourado A. (2015). Epileptic seizure prediction using relative spectral power features. *Clinical Neurophysiology*.

[15] Gadhoumi K., Lina J., Gotman J. (2012). Discriminating preictal and interictal states in patients with temporal lobe epilepsy using wavelet analysis of intracerebral EEG. *Clinical Neurophysiology*.

[16] Zandi A. S., Tafreshi R., Javidan M., Dumont G. A. (2013). Predicting epileptic seizures in scalp EEG based on a variational bayesian gaussian mixture model of zero-crossing intervals. *IEEE Transactions on Biomedical Engineering*.

[17] Huang N. E., Shen Z., Long S. R. The empirical mode decomposition and the Hilbert spectrum for nonlinear and non-stationary time series analysis.

[18] Hearst M. A., Dumais S. T., Osuna E., Platt J., Scholkopf B. (1998). Support vector machines. *IEEE Intelligent Systems and their applications*.

[19] Palaniappan R., Mandic D. P. (2007). EEG based biometric framework for automatic identity verification. *The Journal of VLSI Signal Processing Systems for Signal, Image, and Video Technology*.

[20] Brunner C., Naeem M., Leeb R., Graimann B., Pfurtscheller G. (2007). Spatial filtering and selection of optimized components in four class motor imagery EEG data using independent components analysis. *Pattern Recognition Letters*.

[21] Roberts S. J., Husmeier D., Rezek I., Penny W. (1998). Bayesian approaches to gaussian mixture modeling. *IEEE Transactions on Pattern Analysis and Machine Intelligence*.

[22] Mormann F., Kreuz T., Rieke C. (2005). On the predictability of epileptic seizures. *Clinical Neurophysiology*.

[23] Moody G. B., Mark R. G., Goldberger A. L. PhysioNet: Physiologic signals, time series and related open source software for basic, clinical, and applied research.

[24] Kemp B., Olivan J. (2003). European data format 'plus' (EDF+), an EDF alike standard format for the exchange of physiological data. *Clinical Neurophysiology*.

[25] Niazi I. K., Jiang N., Tiberghien O., Nielsen J. F., Dremstrup K., Farina D. (2011). Detection of movement intention from single-trial movement-related cortical potentials. *Journal of Neural Engineering*.

[26] Ang K. K., Chin Z. Y., Zhang H., Guan C. Filter Bank Common Spatial Pattern (FBCSP) in brain-computer interface.

[27] Huang N. E., Shen Z., Long S. R. The empirical mode decomposition and the Hilbert spectrum for nonlinear and non-stationary time series analysis. In of the Royal Society of London A: mathematical.

[28] Pachori R. (2008). Discrimination between ictal and seizure-free EEG signals using empirical mode decomposition. *Research Letters in Signal Processing*.

[29] Sulaiman N., Taib M. N., Mohd Aris S. A., Abdul Hamid N. H., Lias S., Murat Z. H. Stress features identification from EEG signals using EEG Asymmetry & Spectral Centroids techniques.

[30] Güler İ., Kiymik M. K., Akin M., Alkan A. (2001). AR spectral analysis of EEG signals by using maximum likelihood estimation. *Computers in Biology and Medicine*.

[31] Lan K., Simon F., Song W., Athanasios V., Richard C. Self-adaptive pre-processing approach for big data stream classification of iot environmental sensor monitoring.

